# Effects of expanded adverse childhood experiences including school bullying, childhood poverty, and natural disasters on mental health in adulthood

**DOI:** 10.1038/s41598-024-62634-7

**Published:** 2024-05-26

**Authors:** Natsu Sasaki, Kazuhiro Watanabe, Yoshiaki Kanamori, Takahiro Tabuchi, Takeo Fujiwara, Daisuke Nishi

**Affiliations:** 1 Department of Mental Health, Graduate School of Medicine, The University of Tokyo, 7-3-1 Hongo, Bunkyo-ku, Tokyo, 1130033 Japan; 2 Department of Public Health, Kitasato University School of Medicine, Sagamihara, Japan; 3 Department of Psychiatric Nursing, Graduate School of Medicine, The University of Tokyo, Tokyo, Japan; 4 Cancer Control Center, Osaka International Cancer Institute, Osaka, Japan; 5 The Tokyo Foundation for Policy Research, Tokyo, Japan; 6 Department of Public Health, Tokyo Medical and Dental University, Tokyo, Japan

**Keywords:** Maltreatment, Depression, Anxiety, Life-course, Public health, Medical research, Neurology, Risk factors

## Abstract

The study aimed to examine the association of expanded adverse childhood experiences (ACEs) with psychological distress in adulthood. The data from nation-wide online cohort was used for analysis. Community dwelling adults in Japan were included. The ACEs was assessed by 15 items of ACE-J, including childhood poverty and school bullying. Severe psychological distress was determined as the score of Kessler 6 over 13. Multivariable logistic regression analysis was conducted, by using sample weighting. A total of 28,617 participants were analyzed. About 75% of Japanese people had one or more ACEs. The prevalence of those with ACEs over 4 was 14.7%. Those with ACEs over 4 showed adjusted odds ratio = 8.18 [95% CI 7.14–9.38] for severe psychological distress. The prevalence of childhood poverty was 29% for 50–64 year old participants and 40% of 65 or older participants. The impact of childhood poverty on psychological distress was less than other ACEs in these age cohorts. Bullying was experienced 21–27% in young generations, but 10% in 65 or older participants. However, the impact on psychological distress in adulthood was relatively high in all age groups. ACEs have impacted mental health for a long time. Future research and practice to reduce ACEs are encouraged.

## Introduction

Adverse childhood experiences (ACEs) are traumatic events that children and adolescents under 18 years of age have experienced^1^. People with a history of ACEs are at greater risk of deterioration in physical and mental health^2,3^, and ultimately premature mortality^4,5^. The cumulative effect of a diverse range of ACEs can impact health outcomes in adulthood and far beyond from life-course perspectives^6,7^. Studies increasingly establish evidence that ACEs leads to develop mental health issues, including depression, substance misuse, and suicide^8–11^. A World Health Organization (WHO) study revealed that ACEs were associated with all lifetime DSM–IV disorders worldwide^12^. These associations are explained by both neurobiological development and external factors^13^. For example, early life exposure to chronic stress causes greater activation of the hypothalamic-pituitary-adrena (HPA) axis, and high levels of inflammation, resulting in deficits of cognitive and affective functioning and increased allostatic load^14,15^. ACEs also lead to impaired social functioning, such as lack of social support^16–18^.

ACEs traditionally included childhood maltreatment and household dysfunction. For example, physical neglect, characterized by a caregiver's failure to provide for a child's basic physical needs. Recently, the concept has been expanded to include community-level and social factors, such as school bullying and economic hardship^19–23^. Furthermore, ACEs can differ by race, culture, and era^19,22^. An ACE scale for the Japanese context (ACE-J) has been developed to assess expanded ACEs reflecting the Japanese situation^7^ and its potential benefit of use is to capture the influence of expanded and culturally familiar ACEs on outcomes. For example, Japan has a high prevalence of both school bullying victimization and natural disasters compared to other countries^24,25^. Childhood poverty is also considered an important factor as a root cause of ACEs from life-course perspective^26^. Although the relationship between the conventional ACEs and mental health issues in adulthood have been well investigated, the more recent potential constructs of ACEs should be further explored^27^.

Few studies have examined long-term effects of a wide range of ACEs in Asians. In Japan, the association of conventional ACEs with mental health was last reported in 2011^28^. A scoping review about different types of ACEs from articles (n = 1281) showed that less articles reported expanded ACEs (e.g., household financial hardship; 18%, victimization by peers; 10%, exposure to natural disasters; 2%)^23^. Besides, over 60% of the articles related to ACEs published from United States^23^. This disproportionate evidence motivates researchers to examine long-term impact mental health by expanded ACEs in various countries. The understanding of its association in ethnic groups of a particular culture can lead to the development and implementation of locally sensitive countermeasures^29^. Moreover, empirical evidence about the association of the expanded concept of ACEs on mental health would benefit specifically to evaluate the impacts of each ACE.

The aim of this study was thus to examine the association of expanded ACEs with mental health in adulthood in Japanese community sample. The impact of childhood poverty and school bullying on mental health was investigated, considering demographic indicators.

## Methods

### Research design

For this research, we utilized data from the Japan COVID-19 and Society Internet Survey (JACSIS), an ongoing nationwide online cohort study conducted in Japan^30^. JACSIS cohort study began in August 2020. The JACSIS included community-dwelling individuals aged 15–79 years. The baseline sample of JACSIS was collected in 2020, consisting of 28,000 participants. In 2022, a follow-up survey was conducted for the participants from 2020 survey or 2021 survey, and new participants were also invited. This resulted in a total of 32,000 participants in the 2022 survey. This study employed a cross-sectional design using the JACSIS 2022 data, which was collected in September 2022.

### Participant recruitment

To recruit participants, we utilized email messages to request survey participation from a research panel maintained by Rakuten Insight, Inc. This private company have information about over 2.2 million individuals aged 15 to 79 years with diverse sociodemographic backgrounds, representing the national population across all 47 prefectures of Japan. We employed a simple random sampling method based on sex, age, and prefecture category in accordance with the official Japanese demographic composition as of October 1, 2019, to select potential participants. Those who agreed to participate were provided access to a designated website. Participants had the option to skip questions or discontinue the survey at any point.

### Data quality management

To ensure the validity of the data, we excluded respondents who exhibited discrepancies or provided artificial/unnatural responses. Specifically, we used three question items to identify such responses: "Please choose the second from the bottom," "choosing positive in all of a set of questions for using drugs," and "choosing positive in all of a set of questions for having chronic diseases." A total of 3,370 respondents were found to have provided such responses and were subsequently excluded from the study.

### Participants

Our study included community-dwelling individuals in Japan who were over 18 years old and had complete data. Participants who were under 18 years old were excluded (n = 13).

### Measurement variables

#### Expanded adverse childhood experiences

We assessed Adverse Childhood Experiences (ACEs) using the Adverse Childhood Experiences Japanese version (ACE-J) questionnaire^7^. The ACE-J questionnaire was developed to measure individuals' exposure to various adversities during their childhood in Japan. For example, incarcerated household member was excluded, reflecting Japanese culture. Each category of adversity was represented by a single item, except for parental loss, which included both parental death and divorce or separation. In addition to the CDC-Kaiser ACE questionnaire^1^, the ACE-J included, childhood poverty, overcontrol, school bullying, hospitalization due to chronic disease, and exposure to life-threatening natural disasters. The ACE-J questionnaire consisted of a total of 15 items (parental death, parental divorce, mental illness in the household, substance abuse in the household, mother treated violently, physical abuse, physical neglect, emotional abuse, emotional neglect, childhood poverty, overcontrol [“I always felt suffocated because my parents did not respect my opinion”], school bullying, sexual abuse, hospitalization due to chronic disease, natural disaster), and participants were asked whether they had experienced each adversity before the age of 18. The response options were "Yes" or "No." One item related to emotional neglect was a reversed question, specifically assessing whether participants felt loved by their parents. To calculate the total number of ACEs experienced, the score of the reversed item was reversed, and the summed score of all ACE items was used. The ACE-J questionnaire was not validated in publication.

#### Psychological distress

Psychological distress refers to a broad range of emotional and psychological symptoms or experiences that can cause discomfort, suffering, or impairment in daily functioning.　Psychological distress was measured by The Kessler Psychological Distress Scale (K6), which has been widely used and is preferred for screening for any DSM-IV mood or anxiety disorder. K6 includes six items that measure the frequency of psychological distress symptoms experienced by participants over the past 30 days^31^. Participants provide responses on a scale ranging from 0 (none of the time) to 4 (all the time). Previous studies have reported satisfactory internal reliability and validity for Japanese version of K6, showing that performance in areas under receiver operating characteristic curves (AUCs) was 0.94 detecting DSM-IV mood and anxiety disorders^32^. K6 scores over 13 are regarded as a serious mental distress^31,33,34^. Prevalence of people with over 13 scores of K6 was reported 4% in Japan^35^.

#### Demographic characteristics

The sociodemographic characteristics of the participants were assessed, including age, sex, educational attainment (categorized as less than high school, vocational/college, undergraduate, graduate or over), marital status (categorized as married, single/divorced), household income (categorized as < 3 million yen, 3–5 million yen, 5–8 million yen, 8–10 million yen, over 10 million yen, or no response/unknown), and working status (categorized as paid work, no paid work, or students).

### Statistical analyses

First, the descriptive statistics were estimated. These included the prevalence of ACEs and severe psychological distress and the coexistence of the ACEs. To address a potential sampling bias due to the internet survey, a propensity score for participation in the internet survey was calculated. We utilized a demographic distribution of a national paper-based survey, the Comprehensive Survey of Living Conditions of People on Health and Welfare (CSLCPHW). Using sex and age group stratifications (sex × age groups = 14 strata), we calculated the propensity score separately for each stratum. The mean of the score was group-mean centered and was set to 1.0 within each stratum. Residential area, marital status, education, home-ownership (household), self-rated health and smoking status, which were available both CSLCPHW and JACSIS, were used for the model to calculate the propensity scores. The inversed propensity score was used as the sampling weight for the calculation of the prevalence of the ACEs and psychological distress. The difference in the prevalence of ACEs among stratified categories (sex and age groups) was tested using a chi-square test. Also, the summed number of ACEs was tested using a t-test in sex and one-way analysis of variance (ANOVA) in age category. The coexistence of the ACEs was presented as a matrix.

For the main analysis, the associations of ACEs with severe psychological distress were assessed by using logistic regression analysis, adjusted by age, sex, marital status household income, work, and educational attainment. The sampling bias was also adjusted by the inversed propensity score. Additionally, the subgroup analyses were conducted stratified by sex and age categories. The statistical significance for all analyses in this study is set at 0.05 (two-tailed), and 95% CIs were calculated. SPSS 28.0 (IBM Corp., Armonk, NY, USA) Japanese version was used.

### Ethics approval and consent to participate

The study was reviewed and approved by the Research Ethics Committee of Graduate School of Medicine/Faculty of Medicine, The University of Tokyo (no. 2020336NI-(3)) and by the Research Ethics Committee of the Osaka International Cancer Institute (no. 20084). All methods were carried out in accordance with the Declaration of Helsinki.

### Informed consent

Online informed consent was obtained from all participants with full disclosure and explanation of the purpose and procedures of this study. The panelists had the option to not respond to any part of the questionnaire and the option to discontinue participation in the survey at any point.

## Results

A total of 28,617 community dwelling people was included in the analysis. The participants’ characteristics are presented in Table 1. The mean age was 48 years old (standard deviation [SD] = 17.1). Majority demographics included those who were married (62%), with undergraduate level of educational attainment (46%), and with paid work (65%).

**Table 1:** Participants’ characteristics (N = 28,617).

Items	N (%)
Sex	
Male	13,993 (48.9)
Female	14,624 (51.1)
Age	
18–34 years old	7666 (26.8)
35–49 years old	8258 (28.9)
50–64 years old	6163 (21.5)
65 or older	6530 (22.8)
Marital status	
Married	17,793 (62.2)
Single/divorced	10,824 (37.8)
Educational attainment	
Less than high school	7967 (27.8)
College	6131 (21.4)
Undergraduate	13,008 (45.5)
Graduate over	1511 (5.3)
Household income	
< 3 million yen	4751 (16.6)
3–5 million yen	6200 (21.7)
5–8 million yen	6533 (22.8)
8–10 million yen	2616 (9.1)
Over 10 million yen	2904 (10.1)
No response/unknown	5613 (19.6)
Work	
Paid work	18,693 (65.3)
No paid work	8894 (31.1)
Students	1030 (3.6)

SD: standard deviation.

The sample weighted prevalence of expanded ACEs is presented in Table 2. The overall prevalence of expanded ACEs was varied from lowest (physical neglect = 3.2%) to highest (emotional neglect = 38.5%). The mean of summed number of ACEs was 1.75 (SD = 1.94). The histogram of the summed number of ACEs is presented in Supplementary Fig. 1. The prevalence of those with ACEs over 4 was 14.7%. The prevalence of childhood poverty and school bullying was 26.3% and 20.8%, respectively.

**Table 2:** Sample weighted prevalence of Adverse Childhood Experiences (ACEs) measured by ACE- J in community dwelling Japanese population, stratified by sex and age (N = 28,617).

	ACEs	Overall (N = 28,617)	Sex			Age (years old)				
			Female (n = 14,625)	Male (n = 13,992)	Difference	18–34 (n = 7475)	35–49 (n = 8449)	50–64 (n = 6091)	65 or older (n = 6602)	Difference
		%	%	%	*p* value	%	%	%	%	*p* value
1	Parental death	10.8	10.6	11.0		4.9	6.6	12.4	21.3	**
2	Parental divorce	10.7	11.9	9.6	**	15.4	11.2	8.2	7.2	**
3	Mental illness in the household	4.4	5.0	3.7	**	7.7	4.7	2.7	1.8	**
4	Substance abuse in the household	6.8	6.9	6.6		6.1	7.8	7.9	5.2	**
5	Mother treated violently	8.7	9.5	7.8	**	5.9	9.3	11.5	8.5	**
6	Physical abuse	3.8	4.1	3.6	*	4.6	4.2	4.0	2.3	**
7	Physical neglect	3.2	3.3	3.2		3.1	2.9	3.3	3.8	*
8	Emotional abuse	12.8	16.5	9.0	**	14.1	15.3	15.1	6.1	**
9	Emotional neglect	38.5	34.8	42.3	**	45.9	45.7	36.6	22.5	**
10	Childhood poverty	26.3	25.5	27.1	*	16.5	22.8	28.9	39.5	**
11	Overcontrol	15.5	19.2	11.5	**	14.5	17.5	18.5	11.0	**
12	School bullying	20.8	23.3	18.2	**	21.4	27.3	23.0	9.7	**
13	Sexual abuse	4.4	6.9	1.8	**	4.5	4.8	4.5	3.8	*
14	Hospitalization due to chronic disease	4.8	4.4	5.3	**	3.6	3.6	4.5	8.2	**
15	Natural disaster	3.5	2.9	4.1	**	4.0	2.9	2.7	4.3	**

		Mean (SD)	Mean (SD)	Mean (SD)		Mean (SD)	Mean (SD)	Mean (SD)	Mean (SD)	
	The summed number of ACEs	1.75 (1.94)	1.85 (2.06)	1.65 (1.81)	**	1.72 (1.96)	1.87 (2.03)	1.84 (2.01)	1.55 (1.73)	**
	Number of ACEs	%	%	%		%	%	%	%	
	ACE 0	25.5	27.1	23.7	**	24.7	23.3	25.8	28.7	**
	ACE 1	35.5	31.5	39.7		39.1	36.6	32.6	32.6	
	ACE 2	15.6	15.2	16.0		14.5	14.1	15.4	19.0	
	ACE 3	8.8	9.2	8.4		7.3	9.0	9.5	9.5	
	ACE 4+	14.7	17.0	12.2		14.4	17.0	16.6	10.1	

*p < 0.05, **p < 0.001.

SD: standard deviation.

The difference of each category of sex and age was tested by chi-square test. The each difference in the category of ACE 0, 1, 2, 3, and 4+ was also tested by chi-square test. The difference of the mean of summed number of ACEs was tested by t-test in sex and one-way ANOVA in age category.

For sexual difference, the mean of the summed number of ACEs was larger for females (1.85 vs 1.65; p < 0.001). Sexual abuse was particularly experienced more in female populations (6.9% vs 1.8%). For age difference, the mean of the summed number of ACEs was highest in age 35–49 years old (1.87 [SD 2.03]). Those over 65 years old showed lowest score of ACEs (1.55 [SD 1.73]). Among those over 65 years old, the prevalence of parental death (21%) and childhood poverty (40%) were higher than other age category (p < 0.001), and parental divorce (7%) was lower (p < 0.001).

Table 3 shows the prevalence of severe psychological distress (K6 ≥ 13), which was adjusted for weighed scores. The overall prevalence was 10.4%. The prevalence of severe psychological distress increased as the number of ACEs increased. The highest prevalence of high distress was observed in those with ACEs over 4 in ages 18–34 years old (40.7%). In the group with the same number of ACEs, younger groups showed significantly high prevalence of severe distress compared to older groups (p < 0.001). The significant group difference of sex was not shown in the group with 2 or more ACEs.

**Table 3:** Sample weighted prevalence of high psychological distress (K6 ≥ 13) in community dowelling Japanese population (N = 28,617).

	Overall (N = 28,617)	Sex			Age				
		Female (n = 14,626)	Male (n = 13,993)	Difference	18–34 years old (n = 7476)	35–49 years old (n = 8451)	50–64 years old (n = 6090)	65 or older (n = 6602)	Difference
	%	%	%	*p* value	%	%	%	%	*p* value
Overall	10.4	11.3	9.4	< 0.001	17.6	12.3	7.1	2.6	< 0.001
Number of ACEs									
0	4.3	5.1	3.4	< 0.001	9.2	4.2	2.6	1.1	< 0.001
1	7.6	8.3	7.1	0.017	13.2	8.2	5.2	1.5	< 0.001
2	10.6	10.3	10.9	0.530	18.8	15.3	6.9	1.9	< 0.001
3	13.8	14.4	13.1	0.359	21.9	18.5	10.7	3.8	< 0.001
4+	25.1	25.8	24.1	0.200	40.7	26.3	15.9	11.1	< 0.001

The each difference in the category of ACE was tested by chi-square test.

The result of the associations of ACEs with severe psychological distress is presented in Table 4, using a logistic regression analysis. Almost all individual ACEs, except parental death, were significantly associated with high distress in adulthood in adjusted model (odds ratio ranging from 1.23 to 4.01). The adjusted odds ratio of school bullying, hospitalization due to chronic disease, and natural disaster was 3.04, 2.67, and 2.66; respectively. The odds of high distress increased as the number of ACEs increased; those with ACEs over 4 showed adjusted OR = 8.18 [95% CI: 7.14–9.38].

**Table 4:** Associations of Adverse Childhood Experiences (ACEs) measured by ACE- J with high psychological distress (K6 ≥ 13) in community dwelling Japanese population (N = 28,617).

	ACEs	Crude		Adjusted^a^	
		OR	95% CI	OR	95% CI
1	Parental death	0.82	0.72–0.94	1.23	1.06–1.42
2	Parental divorce	1.63	1.45–1.84	1.28	1.14–1.45
3	Mental illness in the household	3.37	2.94–3.85	2.57	2.23–2.96
4	Substance abuse in the household	2.62	2.32–2.97	2.56	2.25–2.91
5	Mother treated violently	2.15	1.92–2.41	2.40	2.13–2.70
6	Physical abuse	4.01	3.49–4.60	3.65	3.16–4.22
7	Physical neglect	4.19	3.59–4.90	4.01	3.40–4.73
8	Emotional abuse	3.70	3.39–4.04	3.49	3.18–3.83
9	Emotional neglect	2.43	2.25–2.62	1.98	1.83–2.15
10	Childhood poverty	1.71	1.57–1.86	2.14	1.95–2.35
11	Overcontrol	3.69	3.39–4.02	3.71	3.39–4.06
12	School bullying	3.45	3.18–3.74	3.04	2.80–3.31
13	Sexual abuse	3.11	2.71–3.57	3.14	2.71–3.63
14	Hospitalization due to chronic disease	2.33	2.02–2.69	2.67	2.29–3.10
15	Natural disaster	2.57	2.20–3.01	2.66	2.25–3.13
	Number of ACEs (ref: 0)				
	1	2.16	1.89–2.46	1.96	1.71–2.24
	2	3.04	2.63–3.52	3.00	2.58–3.48
	3	4.32	3.68–5.07	4.39	3.72–5.17
	4+	8.54	7.48–9.74	8.18	7.14–9.38

OR, odds ratio; CI, confidential interval.

^a^Adjusted by age, sex, marital status, household income, work, educational attainment, and weighting score.

The result of logistic regression analysis which was stratified by sex and age category is shown in Table 5. Adjusted odds ratio was rather higher in male than female in physical neglect and sexual abuse (aOR = 4.68 [3.68–5.94], aOR = 4.05 [3.06–5.36]; respectively). In age category, physical abuse and physical neglect were highly impacted on the prevalence of high distress among those over 65 years old (aOR = 5.60 [2.87–10.93], aOR = 6.27 [3.41–11.55]; respectively), compared to other age group. However, parental death, parental divorce, and childhood poverty showed lower odds among those over 65 years old and 50–64 years old, compare to 18–34 years old and 35–49 years old.

**Table 5:** Associations of Adverse Childhood Experiences (ACEs) measured by ACE- J with high psychological distress (K6 ≥ 13) in community dowelling Japanese population, stratified by sex and age category (N = 28,617).

	ACEs	Sex									
		Prevalence (%)	Female (n = 14,624)				Prevalence (%)	Male (n = 13,993)			
			Crude		Adjusted^a^			Crude		Adjusted^a^	
			OR	95% CI	aOR	95% CI		OR	95% CI	aOR	95% CI
1	Parental death	10.6	0.60	0.48–0.74	0.86	0.69–1.07	11.0	1.10	0.92–1.32	1.69	1.39–2.05
2	Parental divorce	11.9	1.49	1.27–1.75	1.14	0.97–1.35	9.6	1.83	1.54–2.17	1.47	1.22–1.76
3	Mental illness in household	5.0	3.10	2.59–3.71	2.41	2.00–2.91	3.7	3.76	3.06–4.62	2.79	2.25–3.46
4	Substance abuse in household	6.9	2.66	2.25–3.15	2.60	2.18–3.09	6.6	2.57	2.15–3.08	2.50	2.07–3.03
5	Mother treated violently	9.5	2.20	1.89–2.55	2.42	2.07–2.84	7.8	2.09	1.77–2.48	2.35	1.97–2.81
6	Physical abuse	4.1	3.79	3.13–4.59	3.55	2.91–4.33	3.6	4.26	3.48–5.22	3.74	3.02–4.63
7	Physical neglect	3.3	3.55	2.85–4.41	3.48	2.77–4.38	3.2	5.06	4.04–6.34	4.68	3.68–5.95
8	Emotional abuse	16.5	3.78	3.37–4.24	3.69	3.27–4.16	9.0	3.73	3.24–4.31	3.21	2.77–3.72
9	Emotional neglect	34.8	2.73	2.45–3.05	2.31	2.07–2.59	42.3	2.18	1.95–2.44	1.68	1.49–1.89
10	Childhood poverty	25.5	1.76	1.57–1.99	2.00	1.77–2.28	27.1	1.65	1.46–1.87	2.29	2.01–2.62
11	Overcontrol	19.2	3.71	3.31–4.15	3.76	3.34–4.23	11.5	3.78	3.31–4.31	3.65	3.17–4.19
12	School bullying	23.3	3.23	2.89–3.61	2.89	2.58–3.24	18.2	3.71	3.30–4.18	3.29	2.91–3.72
13	Sexual abuse	6.9	2.83	2.40–3.33	2.84	2.39–3.37	1.8	4.20	3.23–5.48	4.05	3.06–5.36
14	Hospitalization	4.4	2.38	1.94–2.93	2.61	2.10–3.24	5.3	2.29	1.88–2.80	2.69	2.17–3.32
15	Natural disaster	2.9	2.04	1.58–2.62	2.05	1.57–2.67	4.1	3.06	2.50–3.75	3.17	2.56–3.93
	Number of ACEs (ref: 0)	27.1					23.7				
	1	31.5	2.14	1.79–2.56	1.95	1.63–2.34	39.7	2.21	1.82–2.68	2.01	1.65–2.45
	2	15.2	2.78	2.27–3.40	2.65	2.15–3.26	16.0	3.36	2.71–4.16	3.44	2.76–4.28
	3	9.2	4.05	3.26–5.04	4.02	3.21–5.02	8.4	4.66	3.68–5.90	4.94	3.87–6.31
	4+	17.0	7.60	6.38–9.05	7.42	6.18–8.90	12.2	9.96	8.15–12.16	9.26	7.52–11.40

	ACEs	Age category									
		Prevalence (%)	18–34 years old (n = 7666)				Prevalence (%)	35–49 years old (n = 8258)			
			Crude		Adjusted^a^			Crude		Adjusted^a^	
			OR	95% CI	aOR	95% CI		OR	95% CI	aOR	95% CI
1	Parental death	4.9	1.72	1.32–2.24	1.77	1.35–2.32	6.6	1.41	1.11–1.80	1.39	1.09–1.78
2	Parental divorce	15.4	1.39	1.17–1.65	1.29	1.08–1.54	11.2	1.41	1.16–1.72	1.34	1.09–1.64
3	Mental illness in household	7.7	2.40	1.96–2.93	2.30	1.88–2.83	4.7	2.95	2.36–3.70	2.78	2.20–3.50
4	Substance abuse in household	6.1	2.73	2.20–3.38	2.62	2.10–3.27	7.8	2.54	2.08–3.11	2.41	1.96–2.97
5	Mother treated violently	5.9	2.32	1.88–2.87	2.30	1.85–2.85	9.3	2.45	2.04–2.96	2.41	1.99–2.92
6	Physical abuse	4.6	3.50	2.76–4.45	3.23	2.53–4.12	4.2	4.20	3.34–5.27	3.95	3.12–4.99
7	Physical neglect	3.1	4.36	3.32–5.71	4.09	3.10–5.39	2.9	3.86	2.93–5.07	3.67	2.77–4.87
8	Emotional abuse	14.1	2.94	2.52–3.43	2.84	2.43–3.33	15.3	3.59	3.11–4.14	3.44	2.96–3.99
9	Emotional neglect	45.9	1.73	1.53–1.96	1.63	1.43–1.85	45.7	2.11	1.85–2.40	2.00	1.75–2.28
10	Childhood poverty	16.5	2.21	1.90–2.56	2.16	1.85–2.52	22.8	2.40	2.08–2.76	2.27	1.95–2.63
11	Overcontrol	14.5	3.55	3.06–4.12	3.54	3.04–4.13	17.5	3.59	3.12–4.13	3.38	2.92–3.91
12	School bullying	21.4	2.63	2.30–3.00	2.62	2.29–3.01	27.3	3.09	2.71–3.52	2.89	2.52–3.30
13	Sexual abuse	4.5	3.09	2.43–3.93	3.16	2.46–4.04	4.8	3.33	2.65–4.18	3.16	2.49–4.01
14	Hospitalization	3.6	3.06	2.38–3.93	2.97	2.30–3.83	3.6	2.90	2.22–3.78	2.46	1.87–3.23
15	Natural disaster	4.0	2.96	2.29–3.83	2.96	2.28–3.86	2.9	3.03	2.32–3.96	2.95	2.25–3.88
	Number of ACEs (ref: 0)	24.7					23.3				
	1	39.1	1.82	1.51–2.20	1.77	1.46–2.15	36.6	2.17	1.72–2.75	2.08	1.64–2.63
	2	14.5	2.70	2.16–3.37	2.59	2.07–3.25	14.1	3.65	2.83–4.72	3.46	2.68–4.48
	3	7.3	3.46	2.67–4.49	3.50	2.69–4.56	9.0	5.11	3.90–6.71	4.76	3.61–6.28
	4+	14.4	6.75	5.50–8.28	6.44	5.22–7.94	17.0	9.69	7.69–12.21	9.1	7.18–11.54

	ACEs	Prevalence (%)	50–64 years old (n = 6163)				Prevalence (%)	65 or older (n = 6530)			
			Crude		Adjusted^a^			Crude		Adjusted^a^	
			OR	95% CI	aOR	95% CI		OR	95% CI	aOR	95% CI
1	Parental death	12.4	0.85	0.63–1.15	0.90	0.66–1.22	21.3	1.35	0.88–2.05	1.36	0.89–2.09
2	Parental divorce	8.2	1.35	0.98–1.85	1.19	0.85–1.65	7.2	1.30	0.63–2.69	1.23	0.59–2.56
3	Mental illness in household	2.7	3.14	2.14–4.60	2.97	2.00–4.41	1.8	3.36	1.44–7.81	3.08	1.31–7.24
4	Substance abuse in household	7.9	2.46	1.86–3.24	2.15	1.61–2.86	5.2	2.97	1.68–5.25	2.81	1.57–5.03
5	Mother treated violently	11.5	2.31	1.83–2.93	2.18	1.71–2.78	8.5	2.45	1.50–3.99	2.30	1.40–3.78
6	Physical abuse	4.0	2.96	2.16–4.06	2.61	1.89–3.61	2.3	6.30	3.29–12.08	5.60	2.87–10.93
7	Physical neglect	3.3	4.00	2.83–5.64	3.32	2.32–4.74	3.8	6.92	3.85–12.44	6.27	3.41–11.55
8	Emotional abuse	15.1	3.81	3.12–4.64	3.42	2.78–4.21	6.1	5.07	3.28–7.84	4.31	2.73–6.79
9	Emotional neglect	36.6	2.48	2.05–2.99	2.22	1.83–2.69	22.5	2.46	1.69–3.58	2.40	1.64–3.51
10	Childhood poverty	28.9	2.22	1.83–2.69	2.16	1.76–2.64	39.5	1.50	1.04–2.17	1.60	1.08–2.35
11	Overcontrol	18.5	3.70	3.05–4.50	3.29	2.69–4.03	11.0	5.14	3.49–7.59	4.67	3.14–6.96
12	School bullying	23.0	3.55	2.93–4.30	3.04	2.50–3.70	9.7	3.12	1.97–4.92	2.83	1.78–4.51
13	Sexual abuse	4.5	2.88	2.11–3.95	2.48	1.78–3.44	3.8	3.33	1.81–6.14	2.87	1.53–5.39
14	Hospitalization	4.5	2.35	1.69–3.25	2.21	1.58–3.10	8.2	3.75	2.35–5.98	4.04	2.51–6.52
15	Natural disaster	2.7	2.26	1.48–3.45	2.09	1.36–3.23	4.3	1.53	0.71–3.32	1.66	0.76–3.62
	Number of ACEs (ref: 0)	25.8					28.7				
	1	32.6	2.27	1.57–3.27	2.13	1.47–3.08	32.6	1.98	1.11–3.54	2.09	1.16–3.75
	2	15.4	3.49	2.37–5.15	3.16	2.13–4.68	19.0	2.56	1.35–4.86	2.61	1.36–5.01
	3	9.5	5.59	3.74–8.35	5.09	3.39–7.65	9.5	4.13	2.07–8.26	4.41	2.18–8.93
	4+	16.6	9.31	6.60–13.14	8.02	5.64–11.40	10.1	9.23	5.19–16.44	8.89	4.93–16.03

CI, confidential intervals.

^a^Adjusted by age, sex, marital status, household income, work, educational attainment, and weighting score.

Supplementary table 1 shows the relationship between ACEs, which was adjusted for weighted scores. We found high comorbidity of ACEs; for example, those who experienced physical abuse also experienced emotional abuse (75%), overcontrol (68%), and emotional neglect (65%).

## Discussion

This study presented the high prevalence of expanded ACEs in Japan and its strong impact on mental health in adulthood. The mean of summed number of ACEs as measured by expanded ACEs scoring customized for Japanese people was 1.75. The prevalence of those with ACEs over 4 was 14.7% and they significantly showed high odds on severe psychological distress in adulthood, compared to those with none ACE (aOR = 8.18 [95% CI 7.14–9.38]). Childhood poverty showed lower odds among those over 65 years old and 50–64 years old compared to other ACEs. Bullying relatively showed higher odds among all age categories, with some difference of prevalence across age category.

About 75% of participants had one or more ACEs in this study. Reports of worldwide prevalence of ACEs are lower, including 62% in U.S.^36^ and 47% in Europe^37^, by measuring 11 items of ACEs in both studies. A systematic review of a ACE-related study with a large sample reported that a pooled prevalence of individuals with one ACE was 23.5% in Europe and 23.4% in North America, and those with two or more was 18.7% in Europe and 35.0% in North America^38^. However, in expanded ACE study (The Philadelphia Urban ACE Study), a prevalence of 83.2% had at least one ACE and 37.3% experienced four or more ACEs, measured by 14 items with additional stresses including bullying^39^. These studies support our findings of prevalence of expanded ACEs.

Among 15 of the expanded ACEs, emotional neglect, childhood poverty, and bullying showed highest prevalence (39%, 26%, and 21%, respectively). A previous study from 2002–2004 using Japanese data reported that parental death (12%), parental divorce (11%), family violence (10%), and physical abuse (8%) were the most prevalent, but neglect was reported less (2%)^28^. The prevalence of emotional neglect in this present study (26%) may be over reported. When compared to recent studies, the prevalence of psychological neglect was 11.6%^40^. One possible reason was that emotional neglect in our study was measured by an inverse item (i.e., “I felt loved by my parents.”). Reversed items in surveys sometimes cause measurement problems due to misresponses^41^. Since this data was obtained online, the misresponse or careless answer may be more likely to occur compared to in-person interviews. However, based on the finding that there is a significant positive association between the presence of emotional neglect and severe psychological distress, it is possible that emotional neglect is this prevalent in Japan. Possible factors contributing to a high prevalence of emotional neglect might include Japan's traditionally reserved emotional culture (e.g., less expression of positive feelings^42^), insufficient emotional support due to parental employment and household issues, as well as inadequate systems for early detection and protection, potentially resulting in an elevated prevalence rate. The expected level of “loved” for Japanese may be higher than the standard family relationship.

All 15 of the individual ACEs showed the negative impact on mental health, after adjusting covariates. Physical neglect and physical abuse showed highly negative associations (aOR = 4.01, 3.65; respectively). This result was partially consistent with the previous Japanese WHO survey data, which showed parental mental illness and physical abuse strongly affected the onset of mood disorder^28^. With a few exceptions^43^, few paper suggested that physical neglect had a significant impact on mental health; but we should note that those with physical neglect has high comorbidity of ACEs (e.g., childhood poverty, emotional abuse/neglect) in this study. Many studies suggested that emotional abuse and neglect had great impacts on mental health^44–47^. Such comorbidity might strengthen the impact of physical neglect. Consistent with Tzouvara and colleagues (2023), this study demonstrated that all ACEs can negatively impact mental health, and ACEs can manifest differently in different populations^27^.

In this study, school bullying impacted on deteriorated mental health in adulthood among all age categories, although the prevalence of experience was lower in older generations. School bullying have serious and lasting negative impacts on mental health, including depression^48–51^, anxiety^48,50–53^, post traumatic stress disorder (PTSD)^54^, and risk of suicide^51,52^. Japan has a higher prevalence of school bullying compared to most other countries (i.e., Japan 22% vs OECD countries 19%)^55^. This study showed that the prevalence was low in elderly population. The possible reasons for this low prevalence may less awareness, different school dynamics (societal norms), and supportive community functions in old Japanese^56^. A previous study indicated the widely varied exposure to bullying across countries^57^, even in one country, the prevalence may vary from generation to generation. To reduce the prevalence, evidence-based practice is needed to be implemented at school^58^.

Natural disasters as one of ACEs was overall experienced 3.5% and impacted on severe psychological distress in adulthood, except those 65 or older. The findings were in line with the previous studies, demonstrating that when experiencing natural disaster, such as earthquake, heavy rain/snowfall, flood, heatwaves, storm, and/or tsunami, can cause short-term and long-term deterioration in mental health^59–62^. The worldwide climate is rapidly changing and we face the increased risk of natural disaster. Assessing the psychological impact that the experience of natural disaster(s) causes may become increasingly important in the near future, in addition to the effort to avoid children from being exposed such traumatic events.

Childhood poverty was experienced more in older age (40% in 65 or older; 17% in 18–34-year-old group), but the negative impact on mental health was less among the elderly population. This result was in line with a previous study showing that accumulative exposure of the economic hardship impacted mental health, but that negative association was attenuated if they experienced upwards mobility^63^. Many of elderly population in this study experienced childhood poverty, but financial difficulty might not persist and change positively. Even so, we should not ignore the importance of childhood poverty for mental health in adulthood, as significant effects have also been found in older adults. A possible mechanism of the link between childhood poverty and mental health are presented; persistent poverty-related challenging tasks^64^, disengagement coping strategy^65^, diminished spatial short-term memory, and helplessness behaviors^66^. Poverty is not only one of the critical social determinants of health^67^, but also an adversity that should primarily be addressed during childhood, when it has significant implications for neurodevelopment, social development, and behavior. The findings of the present study posed the need to ensure that poverty does not persist among the young generation, who suffered economically in childhood.

Overall, this study showed the cumulative negative impacts of expanded ACEs on psychological distress in Japanese adults, as well as individual adversities. A previous study suggested that a 10% reduction in ACE prevalence could equate to annual savings of 3 million DALYs or $105 billion^38^. Primary prevention, or preventing children from having ACEs is urgent action for public mental health. In addition, childhood maltreatment has consistently been shown to be associated with poor treatment outcome after psycho- or pharmaco-therapy in depression^68^. Trauma-informed care can be one of the important approaches to be implemented for tertiary prevention.

### Limitations

This study has several limitations. First, generalizability of the findings was limited because this was an online cohort study. Although we adopted sample weighting to adjust the bias and examined the prevalence with large number of participants, we should note that the present result may possibly be different from the real data of community dwelling people in Japan. Participants of online survey have access to the internet and motivation to answer the questionnaire with small reward. It is possible that participants with certain demographic characteristics and traits are likely to participate. Second, a recall bias in terms of measuring ACEs was not avoided. Older participants answered less ACEs may underestimate the impact. Third, the definition of school bullying may also vary between younger and older generations. The authors should note that the outcome of this study was obtained self-report questionnaire and it could cause self-reporting bias. Fourth, there are possibly unconsidered/unmeasured confounding factors. Many factors which can impact on mental health during or after COVID-19 have been presented, but not all factors can be comprehensively considered in the analytic model of this study. Fifth, the number of respondents excluded from the analysis due to inappropriate answer was relatively high. It may be possible that this procedure exclude participants with certain response tendencies. Sixth, although K6 has been shown the relationship with clinical outcome and diagnosis, further study which utilize other clinical assessment may need to be conducted in the future. Seventh, the specific age of having adversity is not clear in this study, although the timing may be important in some ACEs. Future research is needed to consider such detailed information and to examine precise mechanism of the associations of ACEs on health.

## Research, policy, and practical implications

Prospective longitudinal study with information about expanded ACEs and clinical diagnosis of mental health disease may be beneficial to suggest the exact impact of ACEs on mental health. Specifically, it is essential to further investigate modifiable childhood factors within the home and school environments to develop effective prevention measures for ACEs through public health policies.

### Supplementary Information

Supplementary Figure 1.

Supplementary Table 1.

## Data Availability

The data used in this study are not available in a public repository because they contain personally identifiable or potentially sensitive patient information. Based on the regulations for ethical guidelines in Japan, the Research Ethics Committee of the Osaka International Cancer Institute has imposed restrictions on the dissemination of the data collected in this study. All data enquiries should be addressed to the person responsible for data management, Dr. Takahiro Tabuchi, at the following e-mail address: tabuchitak@gmail.com.
