# Supplementary figures and images for: Effects of expanded adverse childhood experiences including school bullying, childhood poverty, and natural disasters on mental health in adulthood
Source: Sci Rep. 2024 May 26;14:12015. doi: 10.1038/s41598-024-62634-7 (PMC11128446; doi:10.1038/s41598-024-62634-7)

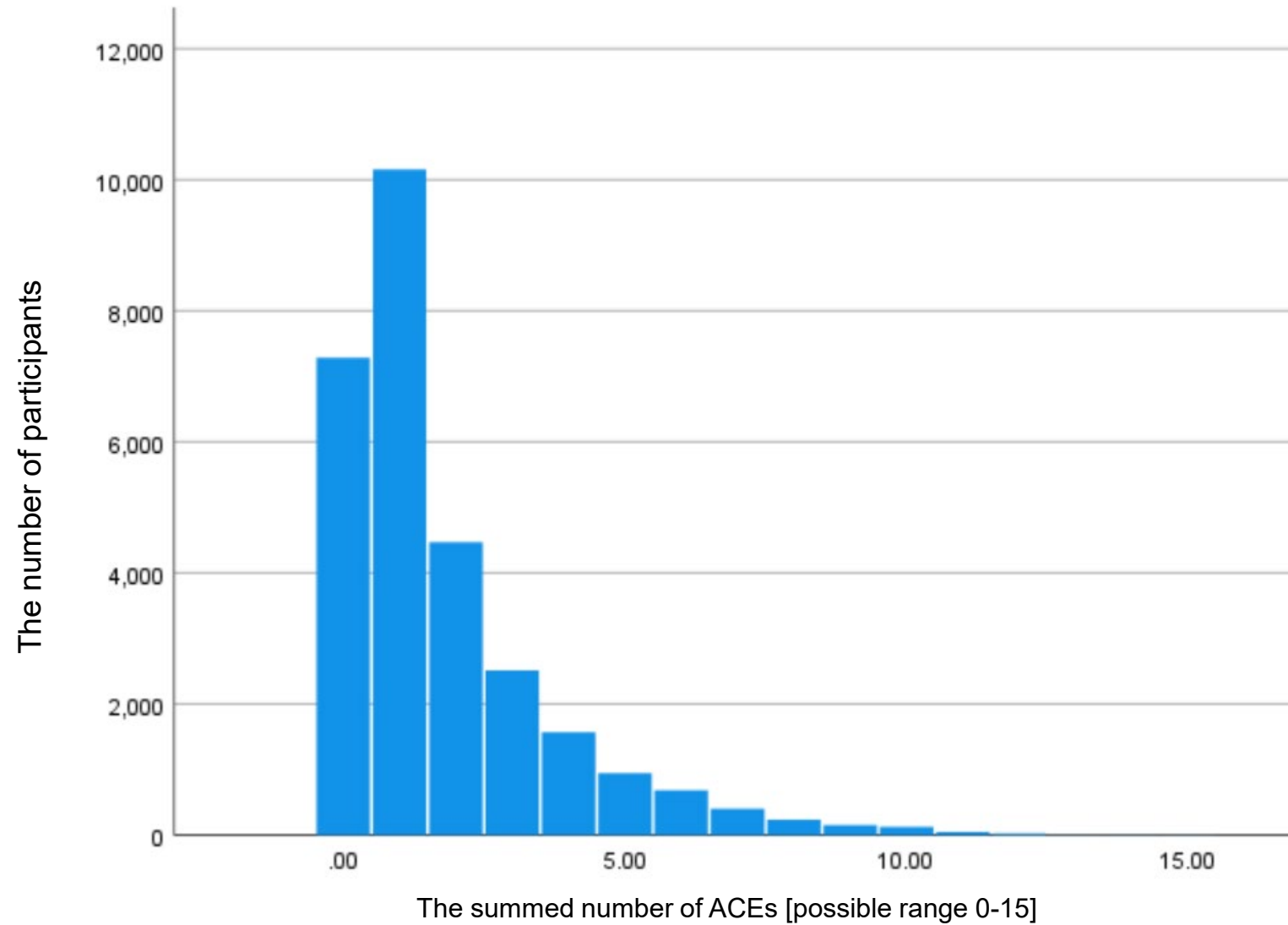

Supplementary figure1. Histogram of the summed number of ACEs.

Supplement: Supplementary file 1 — Supplementary Figure 1. [file 41598_2024_62634_MOESM1_ESM.pdf]
