# Supplementary material for: Effects of expanded adverse childhood experiences including school bullying, childhood poverty, and natural disasters on mental health in adulthood
Source: Sci Rep. 2024 May 26;14:12015. doi: 10.1038/s41598-024-62634-7 (PMC11128446; doi:10.1038/s41598-024-62634-7)
Supplement: Supplementary file 2 — Supplementary Table 1. [file 41598_2024_62634_MOESM2_ESM.docx]

**Supplementary table 1. Sample weighted relationships between categories of Adverse Childhood Experiences (ACEs) (N=28617).**

|  | n (%) exposed to another ACEs | | | | | | | | | | | | | | | |
| --- | --- | --- | --- | --- | --- | --- | --- | --- | --- | --- | --- | --- | --- | --- | --- | --- |
| ACEs | n (%) | 1 | 2 | 3 | 4 | 5 | 6 | 7 | 8 | 9 | 10 | 11 | 12 | 13 | 14 | 15 |
| 1 parental death | 3087 (10.8) | 0 (0) | 432  (14.0) | 247  (8.0) | 336 (10.9) | 400  (13.0) | 209 (6.8) | 257 (8.3) | 417 (13.5) | 907 (29.4) | 1356 (43.9) | 523 (16.9) | 586  (19.0) | 191 (6.2) | 351 (11.4) | 247  (8.0) |
| 2 parental divorces | 3074 (10.7) | 432 (14.1) | 0 (0) | 456 (14.8) | 646  (21.0) | 745 (24.2) | 336 (10.9) | 358 (11.6) | 924 (30.1) | 1257 (40.9) | 1591 (51.8) | 912 (29.7) | 1047 (34.1) | 374 (12.2) | 208 (6.8) | 194 (6.3) |
| 3 Mental illness in the household | 1252 (4.4) | 247 (19.7) | 456 (36.5) | 0 (0) | 403 (32.2) | 354 (28.3) | 215 (17.2) | 228 (18.2) | 563  (45.0) | 568 (45.4) | 588  (47.0) | 492 (39.3) | 551  (44.0) | 201 (16.1) | 181 (14.5) | 149 (11.9) |
| 4 Substance abuse in the household | 1937 (6.8) | 336 (17.3) | 646 (33.3) | 403 (20.8) | 0 (0) | 850 (43.9) | 334 (17.2) | 357 (18.4) | 794  (41.0) | 931  (48.0) | 1230 (63.5) | 785 (40.5) | 805 (41.5) | 304 (15.7) | 215 (11.1) | 218 (11.2) |
| 5 Mother treated violently | 2492 (8.7) | 400 (16.1) | 745 (29.9) | 354 (14.2) | 850 (34.1) | 0 (0) | 604 (24.2) | 377 (15.1) | 1083 (43.5) | 1151 (46.2) | 1440 (57.8) | 1131 (45.4) | 1036 (41.6) | 365 (14.6) | 243 (9.8) | 207 (8.3) |
| 6 Physical abuse | 1095 (3.8) | 209 (19.1) | 336 (30.7) | 215 (19.6) | 334 (30.5) | 604 (55.2) | 0 (0) | 316 (28.9) | 819 (74.8) | 714 (65.2) | 561 (51.2) | 742 (67.8) | 629 (57.4) | 254 (23.2) | 153  (14.0) | 126 (11.5) |
| 7 Physical neglect | 925 (3.2) | 257 (27.8) | 358 (38.7) | 228 (24.6) | 357 (38.6) | 377 (40.7) | 316 (34.1) | 0 (0) | 578 (62.4) | 619 (66.8) | 670 (72.4) | 596 (64.4) | 484 (52.3) | 200 (21.6) | 167  (18.0) | 138 (14.9) |
| 8 Emotional abuse | 3665 (12.8) | 417 (11.4) | 924 (25.2) | 563 (15.4) | 794 (21.7) | 1083 (29.6) | 819 (22.4) | 578 (15.8) | 0 (0) | 2235 (61.0) | 1680 (45.9) | 2502 (68.3) | 1921 (52.4) | 583 (15.9) | 339 (9.3) | 246 (6.7) |
| 9 Emotional neglect | 11006 (38.5) | 907 (8.2) | 1257 (11.4) | 568 (5.2) | 931 (8.5) | 1151 (10.5) | 714 (6.5) | 619 (5.6) | 2235 (20.3) | 0 (0) | 2624 (23.8) | 2612 (23.7) | 2481 (22.5) | 589 (5.4) | 488 (4.4) | 358 (3.3) |
| 10 Childhood poverty | 7528 (26.3) | 1356 (18.0) | 1591 (21.1) | 588 (7.8) | 1230 (16.3) | 1440 (19.1) | 561 (7.5) | 670 (8.9) | 1680 (22.3) | 2624 (34.9) | 0 (0) | 2167 (28.8) | 2439 (32.4) | 656 (8.7) | 640 (8.5) | 461 (6.1) |
| 11 Overcontrol | 4421 (15.4) | 523 (11.8) | 912 (20.6) | 492 (11.1) | 785 (17.8) | 1131 (25.6) | 742 (16.8) | 596 (13.5) | 2502 (56.6) | 2612 (59.1) | 2167 (49.0) | 0 (0) | 2156 (48.8) | 577 (13.1) | 425 (9.6) | 284 (6.4) |
| 12 School bullying | 5946 (20.8) | 586 (9.9) | 1047 (17.6) | 551 (9.3) | 805 (13.5) | 1036 (17.4) | 629 (10.6) | 484 (8.1) | 1921 (32.3) | 2481 (41.7) | 2439 (41.0) | 2156 (36.3) | 0 (0) | 681 (11.5) | 527 (8.9) | 356  (6.0) |
| 13 Sexual abuse | 1264 (4.4) | 191 (15.1) | 374 (29.6) | 201 (15.9) | 304 (24.1) | 365 (28.9) | 254 (20.1) | 200 (15.8) | 583 (46.1) | 589 (46.6) | 656 (51.9) | 577 (45.6) | 681 (53.9) | 0 (0) | 202  (16.0) | 160 (12.7) |
| 14 Hospitalization due to chronic disease | 1384 (4.8) | 351 (25.4) | 208  (15.0) | 181 (13.1) | 215 (15.5) | 243 (17.6) | 153 (11.1) | 167 (12.1) | 339 (24.5) | 488 (35.3) | 640 (46.2) | 425 (30.7) | 527 (38.1) | 202 (14.6) | 0 (0) | 181 (13.1) |
| 15 Natural disaster | 997 (3.5) | 247 (24.8) | 194 (19.5) | 149  (15.0) | 218 (21.9) | 207 (20.8) | 126 (12.7) | 138 (13.9) | 246 (24.7) | 358 (35.9) | 461 (46.3) | 284 (28.5) | 356 (35.7) | 160 (16.1) | 181 (18.2) | 0 (0) |
